# Clinical characteristics and outcomes among patients with COVID-19: A single-center retrospective observational study from Marj, Libya

**DOI:** 10.15537/smj.2022.43.9.20220343

**Published:** 2022-09

**Authors:** Aimen M. Khalifa, Fatimah A. Nouh, Farag A. Elshaari

**Affiliations:** *From the Department of Medicine (Khalifa), Faculty of Medicine, University of Benghazi - Marj; from the Department of Biochemistry (Nouh, Elshaari), Faculty of Medicine, University of Benghazi; and from the Libyan Center for Biotechnology Research (Elshaari), Benghazi, Libya.*

**Keywords:** Coronavirus, Risk factors, Poor outcome, Comorbid diseases, Libya

## Abstract

**Objectives:**

To describe the clinical characteristics and the contributing factors potentially associated with the poorer outcome in Libyan COVID-19 ICU patients.

**Methods:**

The present work is a retrospective, single-center study, which included 94 COVID-19 patients admitted to the Isolation Department at Marj Hospital from August 21st, 2020 till April 30th, 2021. The patients’ data, including their medical history, clinical manifestations, radiological imaging, and laboratory findings, were obtained from the hospital records.

**Results:**

A higher proportion of the admitted patients were males. The patients’ mean age was 68.29 ± 13.64. The patients came with varying symptoms, but most commonly they were affected by dyspnea, fever, cough, and fatigue. Diabetes was the most common underlying comorbidity; nonetheless, other chronic diseases like hypertension, cardiovascular disease, renal disease, and lung diseases individually affected a significant proportion of patients. Although there was no effect of gender on patients’ outcomes, age had a significant influence on the disease consequences.

**Conclusion:**

There was a strong effect of age on ICU admission and patients’ surviving the illness. Diabetes was the most common underlying comorbid disease in COVID-19 patients. On admission time, inflammatory markers such as CRP, D-dimer, serum ferritin, and LDH, in common, were the most important indicators of poorer prognosis. Male gender, comorbidity, and symptomology adversely affected the rate of admission but not the patient survival.

Since the announcement of the first cases in China, the coronavirus disease-19 (COVID-19) virus Means of spread have caused a swift outbreak that has resulted in a global health crisis.^1^ Despite the higher fatality rate caused by this virus’s relatives such as severe acute respiratory syndrome (SARS) and Middle East respiratory syndrome (MERS), COVID-19 is leading to the loss of a higher number of patients due to the greater number of cases. In May 2021, the number of confirmed cases rose to more than 522 million, accompanied by more than 6 million deaths worldwide. In Libya, the confirmed cases were more than 495,000, and the mortality was more than 6,000 deaths.^2^

Coronavirus disease-19 has a wide spectrum of clinical manifestations, typically ranging from the asymptomatic form or mild upper respiratory tract symptoms to the severe form of the disease that might result in multiorgan failure.^3^ In several studies around the world, it was reported that more males than females were admitted to the hospital because of COVID-19 infection, and the most common COVID-19 symptoms upon admission were fever, cough, fatigue, and headache.^4-6^ In the more serious cases, the coronavirus infection causes pneumonia, which may lead to the well-studied SARS, which may result in death.^7,8^ Commonly, COVID-19 infection results in a poorer outcome in patients who suffer from chronic diseases. The most common comorbidities associated with the severity of COVID-19 are hypertension and diabetes.^9,10^ However, diabetes undoubtedly gained more attention as the comorbidity associated with coronavirus disease’s undesirable outcome.^11^

A growing body of evidence suggests that there is a link between a variety of factors and prognosis in coronavirus disease cases. In addition to chronic diseases, aged COVID-19 patients have a poorer prognosis and are more likely to be admitted to the intensive care unit (ICU).^3^

A report published by the Chinese Center for Disease Control and Prevention that took in the records of 72,314 cases, revealed increased mortality among sufferers living with diabetes (7.3%) compared to the overall mortality (2.3%) in COVID-19 patients.^1^ In a different report, when infected with SARS-CoV-2, patients who have cardiovascular illness have a dramatically higher risk of death.^12^

The current study was carried out to enhance the prediction of the possible outcome of COVID-19 and to determine whether patients’ history criteria, including age, gender, smoking, and the presence of underlying diseases, could improve such prediction and help personalize management routine. The objective of the current study was to investigate the influence of clinical characteristics, laboratory investigation data, and disease comorbidity on the outcome of SARS-CoV-2 in patients diagnosed with ARDS. The comparison was carried out by contrasting the characteristics of patients who survived the suffering of COVID-19 acute respiratory distress syndrome (ARDS) with those who unfortunately lost the battle against the viral offense and passed away. Hopefully, through this study, we can draw the attention of the medical community to the predicting factors that might be associated with poorer COVID-19 pneumonia outcomes.

## Methods

The current report is merely based on a single-centered observational retrospective study carried out in 2021. The study encompassed the medical records of 94 hospitalized patients, selected out of 282 patients admitted to the Isolation Department, Marj Hospital, Libya during the specified period from August 21, 2020 to April 30, 2021. The ethical approval carrying the number 2021/11 was duly obtained from the appropriate local authority before commencing the study. In addition, for confidentiality issues, all patients’ data was handled using coding numbers, and no decoding was required at any stage.

The Isolation Department admitted 282 cases who presented with ARDS. All the cases were COVID-19 suspected patients, clinically diagnosed on the basis of symptoms, exposure to infection, oxygen saturation of <94%, and confirmed COVID-19 pneumonia. Critically ill patients having multi-organ failure, septic shock, or in need of non-invasive ventilation or renal replacement therapy were admitted to the ICU. The management routine of patients was according to the COVID-19 treatment protocol document prepared by the Libyan Consultation Medical Committee to Combat Coronavirus Epidemic and endorsed by the local health authorities in September 2020.

**Figure 1:**
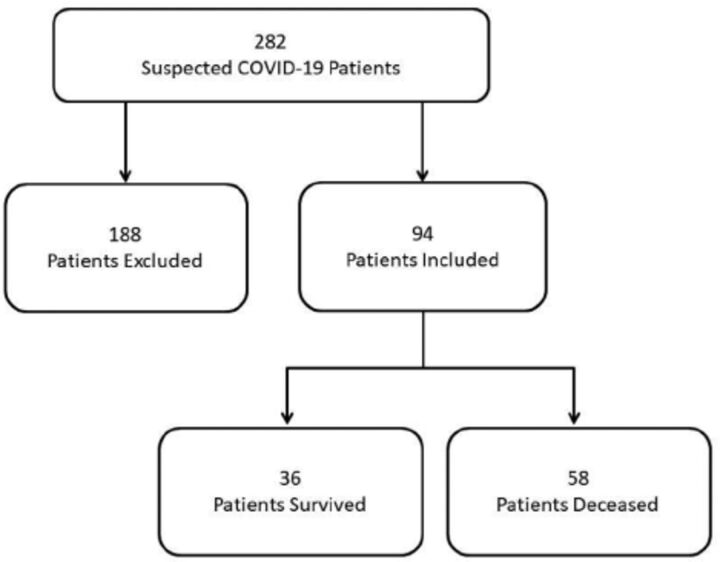
- Flow chart representing the selection steps of patients included in the study.

Patients were selected for the study if they had had a definitive outcome of COVID-19 pneumonia through a positive polymerase chain reaction (PCR) test, positive immunological test, or HRCT chest, and had no alternative diagnosis. Patients were deselected if they matched the exclusion criteria that included the presence of an alternative diagnosis, unconfirmed diagnosis, unknown definitive outcome, or incomplete patient record. Out of the 282 likely patients, 94 patients fit the selection criteria and were appropriately divided into 2 distinct groups. The first group comprised 36 patients who survived the coronavirus intensive care hospitalization. The second group comprised 58 patients that unfortunately expired during ICU or Isolation Department hospitalization (Figure 1).

The selected patients’ data were collected directly from their hospital files. All relevant record information was transferred into a pre-designed data sheet comprised of personal data, signs and symptoms, medical history, and patients’ outcomes.

The targeted laboratory investigations were estimated at the time of admission before any medication that could interfere with the test results was given to the patients. The collected patients’ samples were used to assess the level of activity of blood coagulation and biochemical parameters including liver and renal function (LFT, RFT), creatine kinase (CPK), lactate dehydrogenase (LDH), and electrolytes.

### Statistical analysis

Descriptive statistics were used to describe categorical variables presented by counts and percentages. In contrast, continuous variables were presented as mean and standard deviation. The differences between categorical variables were tested using the Chi-square test, while continuous variables were tested using the Student’s T-test. The statistical significance was set to a *p*-value of <0.05. Statistical analyses were carried out using the Statistical Package for the Social Sciences, version 22 (IBM Corp., Armonk, N.Y., USA).

## Results

A total of 94 admitted patients were clinically diagnosed with COVID-19. Eighty-two (87.2%) were confirmed by chest CT, while 69 (73.4%)cases were confirmed by PCR, and 54 (57.4%) cases by immunoassay. Fifty-seven (60.6%) patients were admitted to the ICU. Fifty-eight (61.7%) patients died during hospitalization, and 36 (38.3%) were clinically cured and discharged from the hospital (Table 1).

**Table 1:** - The proportion of gender, types of symptoms, type of diagnostic procedure, and case outcome.

Factors	n	%
***Gender***		
Male	64	68.1
Female	30	31.9
***Symptoms***		
Dyspnea	94	100
Fever	84	89.4
Cough	78	83
Fatigue	54	57
GIT symptoms	26	27.7
***Chronic diseases***		
Diabetes mellitus	59	62.8
Hypertension	53	56.4
Cardiovascular diseases	41	43.6
Renal disease	11	11.7
Lung disease	7	7.4
Thyroid disease	2	2.1
***Diagnosis***		
Clinical diagnosis	94	100
CT chest	82	87.2
PCR	69	73.4
Immunologically	54	57.4
ICU admission	56	59.6
Deceased patients	58	61.7
Discharged patients	36	38.3

Values are presented as number and percentage (%). PCR: polymerase chain reaction, CT: computed tomography, ICU: intensive are unit, GIT: gastrointestinal tract

Despite the fact that males had a higher admission rate, where 68.1% male and 31.9% female patients were hospitalized within the time of the data collection. Our study demonstrates that there is no effect of gender on patients’ outcome as there was no significant difference between male and female survivors in admitted patients (*p*=0.49). With reference to the symptoms, dyspnea was the most common symptom. Dyspnea was found in 94 (100%) patients, followed by fever (89.4%). Cough was evident in 83%, and 57.4% patients suffered from fatigue. Gastrointetinal tract symptoms were documented in 26 (27.7%) patients. However, no significant differences were seen in mortality regarding any of the noted symptoms (Table 1). With regard to chronic diseases affecting the patients of the study, diabetes was the most common comorbidity in 59 (62.8%) of the admitted patients. Hypertension was the second most seen chronic disease, noted in 53 of the patients [56.4%], followed by cardiovascular (43.6%). In addition, renal disease, lung disease, cardiovascular disease, and cerebrovascular accident (CVA) were the least COVID-19 noted comorbidities, seen in 11.7%, 7.4%, and 13.8% of the patients, respectively (Table 1).

The mean age of all admitted patients was 68.29±13.64 years, and there was a statistically significant difference between the mean age of the survivors (61±12) and that of non-survivors (72±12), where *p*=0.001. The mortality among the admitted cases was found to be higher in patients aged 55 (71.2%) years and more compared to those that were less than 55 (28.6%) years old, where *p*=0.001 (Table 2).

**Table 2:** - Distribution of gender, age, comorbidity and their level of association with COVID-19 outcome.

Characteristics	Total patients		Non-survivors	Survivors	P-values
	n=94 (100%)		n=58 (61.7%)	n=36 (38.3%)
***Gender***					
Male		64 (68.1)	41 (64.1)	23 (53.9)	0.49
Female		30 (31.9)	17 (56.7)	13 (43.3)	
***Age***					
≤55		21 (22.3)	6 (28.6)	15(71.4)	0.001
>55		73 (77.7)	52 (71.2)	21 (28.8)	
Age (mean and SD)			72±12	61±12	0.001
Interval between symptoms and hospitalization			7.5±2.9	8.3±3.9	0.236
***ICU admission***					
Yes	57 (60.6)		51(90)	6(10.0)	
No	37 (39.4)		7(19)	30(81.0)	
***Smoking***					
Yes	7 (7.4)		4 (57.1)	3 (42.9)	0.795
No	87 (92.6)		54 (62.1)	33 (37.9)	
***Diabetes mellitus***					
Yes	59 (62.8)		38 (64.4)	21 (35.6)	0.484
No	35 (37.2)		20 (57.1)	15 (42.9)	
***Hypertension***					
Yes	53 (56.4)		34 (64.2)	19 (35.8)	0.579
No	41 (43.6)		24 (58.5)	17 (41.5)	
***CVD***					
Yes	41 (43.6)		29 (70.7)	12 (29.3)	0.113
No	53 (56.4)		29 (54.7)	24 (45.3)	
**Lung disease**					
Yes	7 (7.4)		3 (42.9)	4 (57.1)	0.286
No	87 (92.6)		55 (63.2)	32 (36.8)	
***Renal disease***					
Yes	11 (11.7)		7 (63.6)	4 (36.4)	0.888
No	83 (88.3)		51 (61.4)	32 (38.6)	
***CVA***					
Yes	13 (13.8)		10 (76.9)	3 (23.1)	0.224
No	81 (86.2)		48 (59.3)	33 (40.7)	
***Comorbidity***					
No comorbidity	15 (16.0)		8 (53.3)	7 (46.7)	0.661
1 comorbidity	21 (22.3)		12 (57.1)	9 (42.9)	
>1 comorbidity	58 (61.7)		38 (65.5)	20 (34.5)	

ICU: intensive care unit, CVD: cardiovascular disease, CVA: cerebrovascular accident

Although, with reference to mortality among diabetic and non-diabetic patients, more diabetic patients died during hospitalization. There was no statistically significant difference between mortality among diabetics (64.4%) and non-diabetics (57.1%) patients. In addition, the means of diabetic random blood sugar (228±107) and hemoglobin A1c (7.5±1.8.8) compared to non-diabetics were higher, but with no statistical significance (*p*>0.05). Furthermore, patients with hypertension, cardiovascular disease, lung disease, CVA, and chronic kidney disease were found to show no statistically significant increase in mortality compared to those with no comorbidity, where *p*>0.05. On the other hand, seven patients were active smokers, but this had no significant effect on mortality, with *p*=0.795 (Table 2). Patients comorbid with more than one chronic disease made up a significant number of the admitted cases. Fifty-eight (61.7%) patients had more than one chronic disease; 34.5% of them survived the corona ordeal; whereas 21 (22.3%) had one comorbid disease, and 42.9% of them survived and were discharged. On the other hand, only 15 (16%) patients were free of any comorbidity, and 46.7% of them survived. The presence of one or more comorbid diseases was not significantly associated with the outcomes of the cases, where *p*=0.661 (Table 2).

Table 3 depicts the mean and the median of the laboratory investigations at the time of admission. The means of CBC, LFT, and RFT were within the normal range, but there were increases in the inflammatory markers such as ESR, CRP, D-dimer, and serum ferritin. Their means and standard deviations were 90.532±31.275, 159.68±115.39, 2647.64 2945.12, and 1266.64±1232.016, respectively.

**Table 3:** - Laboratory investigations and their level of association with coronovirus diseases-19 outcome.

Laboratory characteristics		Total patients	ICU admission	Non-survivors	Survivors	*P*-value
		n=94 (100)	n=57 (60.6)	n=58 (61.7)	n=36 (38.3)	
White blood cells (WBC) (mean±SD)				11.9±6.5	10.8±4.1	0.372
	<4	7 (7.4)	5 (71.4)	6 (85.7)	1 (14.3)	
White blood cells	4-11	43 (45.7)	26 (60.4)	23 (53.5)	20 (46.5)	
	>11	44 (46.8)	26 (59)	29 (65.9)	15 (34.1)	
Hemoglobin (HB) (mean +/-SD)			12.3±2	12.5±2	12.4±2	0.995
Platelet (mean +/-SD)			230±100	217±110	242±75	0.229
Eythrocyte sedimentation rate (mean +/-SD) mm/hr.			95±34	93±33	86±27	0.295
Creactive protein (mean +/-SD) mg/l			177±128	181±128	124±80	0.021
D-dimer (mean +/-SD) ng/ml			3252±3134	3319±3104	1564±2322	0.04
Serum Ferritin (mean +/-SD) ng/ml			1494±1472	1471±1470	935±573	0.039
Lactate dehydrogenase (mean +/-SD) U/L			648±407	645±404	408±187	0.01
B.Glucose (mean +/-)SD mg/dl			212±104	228±107	192±97	0.1
HBA1C (mean +/-SD) %			7.2±1.8	7.5±1.8	7.4±2	0.94
UREA (mean +/-SD) mg/dl			71±42	69±40	60±64	0.363
Creatinine (mean +/-SD) mg/dl			1.7±1.5	1.6±1.4	1.3±1.1	0.289
Alanine aminotransferase (mean +/-SD) U/L			47.3±30	43±27	40±24	0.648
Aspartate aminotransferase (mean +/-SD) U/L			56±50	51±46	45±36	0.495

Values are presented as number and percentages (%).

The means of WBC, HB, platelets, and ESR did not show any significant differences comparing the non-surviving and surviving patients, with *p*>0.05. On the contrary, statistically higher levels were found in the inflammatory markers, CRP (*p*=0.021), D-dimer (*p*=0.04), serum ferritin (*p*=0.039), and LDH (*p*=0.01) in the expired patient group compared to the surviving patients (Table 3). Regarding the symptoms noted in COVID-19 patients included in the current study, dyspnea was the most common symptom, which was seen in 94 (100%) patients, followed by fever, which was seen in 84 (89.4%) patients, followed by cough, which was seen in 78 (83%) patients. Fatigue was seen in 54 (57.4%) patients and GIT symptoms were seen in 26 (27.7%) patients. No significant difference was noted in the mortality between survivors and non-survivors regarding the aforementioned symptoms (Table 4).

**Table 4:** - Clinical symptoms and their level of association with coronovirus diseases-19 outcome.

Clinical symptoms	Total patients	ICU admitted	Non-survivors	Survivors	*P*-value
	n=94 (100%)	n=56 (59.6%)	n=58 (61.7%)	n=36 (38.3%)	
***Fever***					
yes	84 (89.4)	49 (58.3)	49 (58.3)	35 (41.7)	0.051
no	10 (10.6)	8 (80)	9 (90.0)	1 (10)	
***Cough***					
yes	78 (83)	46 (59)	45 (57.7)	33 (42.3)	0.77
no	16 (17)	11 (68.8)	13 (81.3)	3 (13.7)	
***Fatigue***					
yes	54 (57.4)	28 (51.9)	28 (51.9)	26 (48.1)	0.22
no	40 (42.6)	29 (72.5)	30 (75.0)	10 (25)	
***GIT symptoms***					
yes	26 (27.7)	17 (65.4)	15 (57.7)	11 (42.3)	0.621
no	68 (72.7)	40 (58.8)	43 (63.2)	25 (36.8)	

Values are presented as number and percentages (%). ICU: intensive care unit

## Discussion

Corona became a pandemic soon following its outbreak towards the end of 2019. Libya dutifully reported the detection of the first diagnosed corona case in March 2020. Concerning the epidemiological and genetic differences between the inhabitants of various parts of the world, the purpose of this retrospective study was to report the clinical characteristics and potential risk factors associated with the adverse outcome in Libyan patients who had experienced COVID-19 severe acute respiratory syndrome.

Specific diagnosis of COVID-19 has evolved tremendously to undergo a considerable number of changes as further pathophysiological information became obtainable. Currently, the COVID-19 diagnosis is usually validated by a positive PCR test. However, for several apparent reasons, like the limitation of PCR testing capacity being insufficient to properly handle the considerable number of likely patients requiring the test, pressed for other means of high sensitivity testing, these methods, which include sensitive chest CT^13^ and lung computed tomography (CT), have legitimately been added to the diagnostic criteria for Marj Hospital patients. In fact, 87.2% of our patients were chest CT COVID-19 confirmed cases.

Out of the confirmed 94 cases, 64 (68.1%) were male and 30 (31.9%) were female. Our retrospective study revealed that males had a higher risk of eventually developing ARDS and a greater rate of patient admission than females, with a ratio of 2:1. Similar findings were reported in Libya by Nouh et al.^14^ However, there was no significant difference in early mortality comparing the 2 genders. The greater admission rate in observed males was possibly mediated by several contributing factors, including sex hormones and higher expression of coronavirus receptors (ACE 2).^15^

In the current study, age was the most significant risk factor that influenced the disease severity and, thus, was associated with the poorer outcome. This may be satisfactorily explained by the broad spectrum of physiological changes typically affecting the immune system during the aging process. Aging can invariably lead to a progressive decrease in the immune response and, hence, could increase the susceptibility to infection and disease severity.^16^

In our study and that of Nouh et al^14^ among the chronic diseases comorbid with COVID-19, diabetes and hypertension were undoubtedly the most common chronic diseases seen in our patients. Patients with one or more comorbidities had a higher rate of admission. However, regarding disease progression, there is no existing scientific evidence of unfavorable outcomes in COVID-19 patients with comorbidity.^17^ A published report based on a study carried out in Wuhan, China, similarly presented no effect of chronic disease comorbidity on COVID-19 ICU admission and patient survival rate.^4^ On the other hand, similar to a previous study conducted in Saudi Arabia, smoking in our study showed no association either with the admission rate or with the poorer outcome of the patients’ management (ICU admission and death).^18^

The laboratory investigations evidently revealed significant elevations in the tested inflammatory markers (ESR, CRP, D-dimer, and serum ferritin) at the admission time. Furthermore, the inflammatory indicators like CRP, D-dimer, serum ferritin, and LDH were considerably higher in ICU admitted patients and in expired patients compared to those who were ward admitted. These specific findings are consistent with the previously published evidence that suggested that the noticed rise in CRP, D-dimer, and LDH levels could be used as progress indicators of COVID-19 severity.^19^ On the contrary, all other laboratory investigations considered for our admitted patients (WBC, HB, platelets, ESR, RFT, and LFT) had no significant relationship with cases of mortality. Whilst dyspnea was the most common presenting symptom noticed, our patients were also affected by less frequent symptoms, including fever, cough, fatigue, and GIT signs. Nonetheless, all these specific symptoms had no significant effect on the overall case mortality rate reported for our patients. However, in their systematic review, Gold and coworkers reported the need for studies to assess the mechanisms pertinent to the relationship between the severity of COVID-19 ARDS and comorbidity. They came to the conclusion that these studies are critical to understanding this new and evolving disease. We believe that they are also essential for developing a suitable management routine for patients at risk from SARS-Cov-2 and other similar respiratory infections.^20^

### Study limitations

The sample size of the current study was relatively small due to the capacity of the COVID-19 care unit. In this regard, the results and conclusions should be interpreted with caution.

In conclusion, we report a strong effect of the age of corona cases on ICU admission rate and patients’ survival. Diabetes was the most common comorbid disease in the studied COVID-19 patients. At the noted time of admission, inflammatory markers like CRP, D-dimer, serum ferritin, and LDH were the most important indicators of a poor prognosis. Male gender, comorbidity, and symptomatology positively affected the rate of admission but not the survival rate. In addition, smoking had no direct relationship to the poor prognosis. Finally, with somewhat successful efforts to extensively vaccinate the world populations, more studies will be naturally required to follow up on patients with comorbidity to properly assess the level of protection following their survival from the COVID-19 ordeal.
